# Function and development of interneurons involved in brain tissue oxygen regulation

**DOI:** 10.3389/fnmol.2022.1069496

**Published:** 2022-11-24

**Authors:** Daniil P. Aksenov, David A. Gascoigne, Jubao Duan, Alexander Drobyshevsky

**Affiliations:** ^1^Department of Radiology, NorthShore University HealthSystem, Evanston, IL, United States; ^2^Department of Anesthesiology, NorthShore University HealthSystem, Evanston, IL, United States; ^3^Pritzker School of Medicine, University of Chicago, Chicago, IL, United States; ^4^Center for Psychiatric Genetics, NorthShore University HealthSystem, Evanston, IL, United States; ^5^Department of Psychiatry and Behavioral Neuroscience, The University of Chicago, Chicago, IL, United States; ^6^Department of Pediatrics, NorthShore University HealthSystem, Evanston, IL, United States

**Keywords:** vasomotion, fMRI, VIP, NOS, SST, neurovascular coupling

## Abstract

The regulation of oxygen in brain tissue is one of the most important fundamental questions in neuroscience and medicine. The brain is a metabolically demanding organ, and its health directly depends on maintaining oxygen concentrations within a relatively narrow range that is both sufficiently high to prevent hypoxia, and low enough to restrict the overproduction of oxygen species. Neurovascular interactions, which are responsible for oxygen delivery, consist of neuronal and glial components. GABAergic interneurons play a particularly important role in neurovascular interactions. The involvement of interneurons extends beyond the perspective of inhibition, which prevents excessive neuronal activity and oxygen consumption, and includes direct modulation of the microvasculature depending upon their sub-type. Namely, nitric oxide synthase-expressing (NOS), vasoactive intestinal peptide-expressing (VIP), and somatostatin-expressing (SST) interneurons have shown modulatory effects on microvessels. VIP interneurons are known to elicit vasodilation, SST interneurons typically cause vasoconstriction, and NOS interneurons have to propensity to induce both effects. Given the importance and heterogeneity of interneurons in regulating local brain tissue oxygen concentrations, we review their differing functions and developmental trajectories. Importantly, VIP and SST interneurons display key developmental milestones in adolescence, while NOS interneurons mature much earlier. The implications of these findings point to different periods of critical development of the interneuron-mediated oxygen regulatory systems. Such that interference with normal maturation processes early in development may effect NOS interneuron neurovascular interactions to a greater degree, while insults later in development may be more targeted toward VIP- and SST-mediated mechanisms of oxygen regulation.

## Introduction

The regulation of brain tissue oxygen remains a difficult question to address, as its modulatory mechanisms change through development. While early research has predominantly focused on the involvement of excitatory neurons in neurovascular coupling, recent research has sought to elucidate the respective contribution of inhibitory GABAergic interneurons. GABAergic interneurons have a range of important functions in the mature brain, which includes an important contribution to brain tissue oxygen regulation. In order to fully understand brain tissue oxygen dynamics across development, the relative contributions of various types of GABAergic interneurons, along with their corresponding developmental trajectories, must first be understood.

## Inhibitory function of interneurons

Approximately 80–90% of cortical neurons are glutamatergic pyramidal cells, with the remaining 10–20% consisting of inhibitory GABAergic interneurons. The functions of GABAergic interneurons are typically considered to include the direct inhibition of excitatory cells (in either feed-forward or feed-back circuitry) and disinhibition of excitation, *via* the hyperpolarization of other interneurons. As a result, GABAergic interneurons work to spatially and temporally modulate excitability in neuronal networks. This is essential for both basic stimulus integration and higher cortical functions, such as information processing, executive functioning, and learning.

In the mature brain, GABA rapidly induces hyperpolarization in target cells by activating GABAA and GABAB receptors, whereby an influx of chloride ions produces a hyperpolarized state. The result of this hyperpolarization is a significant increase in the relative threshold of excitation required for an action potential to occur in the target cell. This hyperpolarization, therefore, reduces the excitability of neurons.

The inhibitory function of GABAergic interneurons is integral to the notion of excitatory-inhibitory balance (EIB) in the brain, which is typically referred to as the physiological levels of both excitatory and inhibitory activity in the brain. During endogenous or exogenous neuronal activation, the EIB undergoes a dramatic shift toward excitation. On the other hand, during the resting state, the baseline level remains within a narrow range. A permanent or substantial shift in this baseline, typically toward excitation, is associated numerous pathologies, such that minor shifts are thought to participate in the etiology of autism spectrum disorder (Rubenstein and Merzenich, 2003), while much more substantial shifts are characteristic of epilepsy (Staley, 2015).

## Interneurons and brain tissue oxygen regulation

### Interneurons and vasomotion

During periods of increased neuronal activity, the canonical viewpoint credits excitatory pathways for providing the fundamental mechanisms that give rise to neurovascular interactions. For example, nitric oxide, calcium ions, potassium ions, and arachidonic acid metabolites have been thought to stimulate local increases in oxygen delivery (Archer et al., 1994; Attwell et al., 2010; Ross, 2012; Nippert et al., 2018). However, there have also been important, contradictory findings that illustrate the importance of non-excitatory pathways in brain tissue oxygen regulation. These findings include the often occurrence of astrocytic calcium ion projections after the initiation of vasodilation, nitric oxide not being the active signaling molecule in the cerebral cortex, and GABAergic interneurons being essential for the complete elicitation of the hemodynamic response during stimulation (Nippert et al., 2018). It has also been shown that GABA-agonist-induced vasodilation persists even in the presence of nitric oxide synthase inhibitor (Fergus and Lee, 1997). Together, these findings suggest that excitatory pathways are not a complete and sufficient explanation for neurovascular interactions.

It has been previously shown that arterioles carry GABA receptors (Vaucher et al., 2000), and GABAergic terminals from interneurons establish functional contact with arterioles (Cauli et al., 2004; Tremblay et al., 2016). Through this functional connectivity, interneurons have been shown to be essential for the full expression of the hemodynamic response following chemical (Kocharyan et al., 2008), electrical (Kocharyan et al., 2008), optogenetic (Anenberg et al., 2015), and sensory (Aksenov et al., 2019) stimulation as well as during epileptiform discharges (Saillet et al., 2016). In light of this propensity to contribute to the hemodynamic response, interneurons have also been investigated for their contribution to brain tissue oxygen regulation in the resting state.

The resting state is characterized by brain tissue oxygen oscillations (BTOO), which have shown to be necessary for generating sufficient oxygen gradients, to deliver oxygen to brain tissue *via* diffusion that is relatively distant from vessels. Such BTOO result from fluctuations in arteriole tone (Hudetz et al., 1998; Aalkjaer et al., 2011; Mateo et al., 2017) and are an important protection mechanism against hypoxic damage in normal (Doubovikov and Aksenov, 2020) and pathological conditions [e.g., traumatic brain injury and stroke (Li et al., 2021)]. Compared to the hemodynamic response, interneurons can play an even more fundamental role in the regulation of BTOO due to their ability to relay resting state activity to vessels *via* GABA receptors. Indeed, it was shown that blocking GABA-receptors greatly decreases the frequency of BTOO (Aksenov et al., 2022). However, not all interneurons can be responsible for BTOO. It was shown using a patch clamp recording that only a very small subset of interneurons regulates vasodilation (Cauli et al., 2004; Tremblay et al., 2016).

A comprehensive understanding of the neuronal regulation of hemodynamic response and BTOO must consider the developmental differences between adults and neonates. Hypoxia is the primary reason of neonatal death in both humans and animals (Munnich and Kuchenmeister, 2014; Millar et al., 2017), and the mechanisms that regulate brain oxygen levels change significantly with development. The hemodynamic response can be weak in neonates due to diminished neuronal response (Karen et al., 2019; Nourhashemi et al., 2020), which is characterized by low oxygen consumption. It has been shown in a number of studies, that local (i.e., an area of approximately 100 μm^3^) brain tissue oxygen, measured directly with oxygen microelectrodes, is characterized by continual spontaneous oscillations (Manil et al., 1984; Linsenmeier et al., 2016; Aksenov et al., 2018). BTOO in awake neonatal brains demonstrate lower frequencies, ranging from 0 to 7 cycles per minute (cpm) with a peak frequency near 1–2 cpm. Conversely, the adult brain exhibits higher frequencies of BTOO (0–20 cpm), with a peak frequency near 10 cpm. It is known that neonates have a higher tolerance for hypoxia than adults (Singer, 1999), and having both slower oscillations as well as diminished neuronal response likely embodies an important contributing factor for such a difference (Doubovikov and Aksenov, 2020).

### Inhibition and hypoxia

The contribution of the GABAergic system to preventing hypoxia may be integral to understanding the developmental changes of brain tissue oxygen dynamics. The normal development the GABAergic system is characterized by a gradual change in the GABA-driven force, which represents the level of GABA-induced polarization (Tyzio et al., 2008). During early development, GABA can depolarize target neurons in the cortex by eliciting an efflux of chloride ions (Represa and Ben-Ari, 2005; Kirmse et al., 2015). This phenomenon is needed to activate a number of signaling mechanisms that influence dendritic development, cell migration, proliferation, and synaptogenesis (Cellot and Cherubini, 2013; Leger et al., 2020). The interactions between neurons and their supportive microvasculature are underdeveloped during this period (Kozberg and Hillman, 2016) and may be unable to provide sufficient delivery of oxygen during increased neuronal activity (Andreone et al., 2015). Hence, the combined effect of a lack of inhibitory mechanisms and incipient neurovascular coupling exposes the risk of localized hypoxia.

Thus, potential protective mechanisms against localized hypoxia include weak neuronal responses to stimulation and low-frequency spontaneous oxygen oscillations. It was shown that neonatal low-frequency oxygen oscillations support a higher amplitude of oxygen concentration in areas which are distant from the oxygen source (Doubovikov and Aksenov, 2020). Later in development, when neurons and neurovascular interactions are becoming more mature, the inhibitory function of GABA receptors is strengthening until it finally reaches strong adult inhibitory level. At this stage, mature neurons require more efficient oxygen delivery, and as the inhibitory system develops further into adulthood, interneurons emerge as a strong regulator of the total localized neuronal activity. When the activity of surrounding neurons increases, the interneurons are activated in order to directly dilate vessels. In doing so, interneurons can play a key role in preventing localized hypoxia.

### Combined inhibition and regulation of vasomotion

More recently, the involvement of inhibitory interneurons in brain tissue oxygen regulation has become better understood. Evidence suggests, the mechanism by which GABAergic interneurons directly participate in neurovascular coupling involves the hyperpolarization of smooth muscle cells along arterioles (Vaucher et al., 2000). The rapid signaling from interneurons to arterioles, is likely imperative to support neurovascular coupling, especially at the onset of increases in neuronal activity and oxygen demand. While interneurons seem to be heavily involved in neurovascular coupling, it should be noted that different types of GABAergic interneurons are found in the brain, which suggests their involvement in brain tissue oxygen regulation is non-uniform.

Interneurons may also prevent hypoxia indirectly through the inhibition of excitatory neurons. Such inhibition restricts oxygen consumption by preventing excessive neuronal excitation. During action potentials, the required ATP to restore intracellular potassium ion levels is dramatically elevated, leading to the rapid consumption of available oxygen (Ivanov et al., 2015). By preventing excessive excitatory levels, GABAergic interneurons, therefore, prevent hypoxic conditions indirectly *via* inhibition.

The direct and indirect pathways of preventing hypoxia likely work together to prevent hypoxia. This was demonstrated in a recent study which observed transient, severe drops in oxygen concentrations after the administration of a GABA antagonist (Aksenov et al., 2022). By mimicking the debilitation of GABAergic signaling, EIB exhibited a significant shift toward excitation, such that there were intermittent spikes in local field potentials, coupled with dramatic drops of tissue oxygen concentrations below 10 mm Hg. Since these effects were shown without the presence of seizures, these findings support the complex role of GABAergic interneurons in neurovascular coupling and brain tissue oxygen regulation. However, considering the variety of GABAergic interneurons, it remains to be addressed whether or not their contribution to neurovascular interactions is uniform.

### Interneuron subtype specific contributions

The primary types of interneurons discussed below are vasoactive intestinal peptide-expressing (VIP) interneurons, somatostatin-expressing (SST) interneurons, and nitric oxide synthase-expressing (NOS) interneurons, which have varying vasoactive properties (Cauli et al., 2004). VIP interneurons are known to be heavily involved in feedback inhibition of SST interneurons, in the supragranular and infragranular layers of the cortex (Pfeffer et al., 2013). SST interneurons, in contrast, predominantly inhibit non-SST interneurons and the most numerous parvalbumin-expressing (PVB) interneurons preferentially inhibit one another and less so other interneurons (Pfeffer et al., 2013).

Similarly, to the differing roles in circuitry, interneurons also seem to have differing contributions to neurovascular coupling. Importantly, NOS interneurons can be found between excitatory pyramidal cells and the microvasculature, where the release of NO can initiate vasodilation (Cauli et al., 2004). Moreover, NO has shown to induce its vasodilatory effects at a radius of up to 350 μm from its source (Santos et al., 2012), but is likely temporally and spatially confined by the co-release of neuropeptide Y, a potent vasoconstrictive agent. It has been postulated that this co-expression serves to constrict distal microvessels, while inducing proximal vasodilation, to create a local microsphere of increased perfusion (Estrada and DeFelipe, 1998).

In addition, there appears to be a stark contrast between VIP and SST interneurons which have been reported to have a dilating and constricting effect on microvessels, respectively (Cauli et al., 2004). Importantly, the same study also reported that the activation of a single VIP or SST interneuron was sufficient to have the corresponding effect on the immediately surrounding microvasculature, such that VIP and SST interneuron activities directly manipulate vascular tone. Such findings point to the interconnectivity of neurovascular coupling to redirect blood flow from areas of lower neuronal activity to areas of increased neuronal activity, leading to spatially confined functional hyperemia.

It is also important to note that heterogeneity also exists within the expression type of interneuron. For example, VIP interneurons are predominantly vertically oriented bipolar cells in layers II and III of the cortex (Lim et al., 2018), but have also been reported to be multipolar cells in the supragranular layers of the cortex and are transcriptionally different to the VIP bipolar cells due to the co-expression of neuropeptide cholecystokinin (Tremblay et al., 2016). In addition, NOS interneurons can be further divided into two types (type I and II). Type I cells exhibit more intense immunohistochemical markers of NOS characteristics and are distributed throughout cortical layers, while type II are typically found in the supragranular in humans (Duchemin et al., 2012). It is interesting that VIP interneurons are very sensitive to temperature changes, so that increases in the temperature decrease the probability of action potentials (Collins and Ninan, 2021). Perhaps this property is related to local hemodynamic regulation, such that, when experiencing relatively high temperature, VIP neurons do not exacerbate the vasodilatory consequences of the heat itself.

Therefore, given the diversity of interneuron functioning in the mature brain, particularly their involvement in brain tissue oxygen regulation, both at the level of neuronal activity and neurovascular coupling, abnormal development of such neurons can potentially have a range of harmful outcomes.

## Interneuron development

GABAergic interneurons undergo several important developmental milestones. A total of 65% of inhibitory interneurons in the cortex emanate from the neocortical ventricular and subventricular regions of the dorsal forebrain, with the remaining 35% from the ventral forebrain (Letinic et al., 2002; Kelsom and Lu, 2013). These interneurons experience their most intense period of generation and migration from 10 to 25 weeks of gestation (Van Eden and Uylings, 1985; Ganella and Kim, 2014), and their developmental trajectories can continue for as long as 25 years (Arain et al., 2013). During the early stages of development, however, GABAergic signaling undergoes a dramatic shift. Initially, the GABA-driven force, is thought to be excitatory in nature (Fukuda et al., 1998; Kuner and Augustine, 2000; Spitzer, 2010), which then shifts to its characteristic inhibitory functioning once chloride ion transporters are sufficiently developed to reverse the direction of the chloride ion concentration gradient (Ben-Ari, 2002), such that there is a greater concentration outside the cell. The excitatory ability seems to be essential in the early stages of brain development, as GABAergic post-synaptic potentials are functionally active prior to glutamatergic projections (Tyzio et al., 1999; Khazipov et al., 2001). In turn, the early depolarizing function of interneurons is essential for a range of developmental processes including radial and tangential proliferation, neurite growth and synapse formation (Peerboom and Wierenga, 2021). Hence, the normal develop of GABAergic development is essential for optimal maturation and brain functioning.

In line with their heterogeneity in functioning, there are also discrepancies in the development between different types of interneurons. As described previously, VIP and SST interneurons functionally interact with one another, and their developmental trajectories are similarly interconnected. A recent study has been able to contrast the development of VIP and SST interneurons in a mouse model (Collins and Ninan, 2021). The investigators reported VIP interneurons in the adolescent brain exhibits enhanced input signaling with diminished membrane excitability. This phenomenon coincides with elevated inhibitory activity of SST interneurons (Koppensteiner et al., 2019). These findings are in line with previous literature which observed a similar shift in cortical interneuron activity during stimulation. In this study, in the adolescent stage of development (postnatal day 14 in mice) VIP interneurons demonstrated greater selectivity to input signals, with diminished overall response rates, while SST interneurons showed increased excitability from more general sensory stimulation (Kastli et al., 2020). The combination of decreased VIP and increased SST inhibitory signaling seems to lower the disinhibition of pyramidal cells and, in turn, affect their ability to undergo plasticity (Collins and Ninan, 2021). Thus, VIP and SST undergo a dramatic divergence in development, which should be reflected in the changes of neurovascular and brain tissue oxygen regulations. We suggest that VIP interneurons develop together with neuronal responses, so that the increasing specificity of neuronal response accompanies better vasodilation due to the activation of VIP interneurons. Indeed, it was shown that VIP interneurons lose their multi-whisker preference during development (Kastli et al., 2020), which makes their responses more specific and discrete.

On the other hand, NOS interneurons have region-dependent origins and maturation trajectories. Hippocampal NOS interneurons homogenously originate in the medial geniculate eminence (MGE), while neocortical interneurons show a separation between type I and II. Type I NOS interneurons have consistently shown to develop in the MGE (Perrenoud et al., 2012), while type II can originate in both the MGE or caudal ganglionic eminence/entopeduncular regions (Jaglin et al., 2012). Moreover, NOS interneurons are important for a range of early developmental processes. An example is the activation of the NO cGMP pathway, which is essential for neuronal development, synaptic modulation, and learning (Tricoire and Tania, 2012). NOS interneurons are also involved in paracrine signaling for cell differentiation and proliferation (Barnabé-Heider and Miller, 2003), and neuronal excitability (Garthwaite, 2008). Thus, the important periods NOS interneuron development seem to be earlier than that of VIP and SST interneurons. Moreover, NOS interneurons appear to be more heavily involved in general brain development and may not produce stimulus-discrete vasodilation, in contrast to VIP neurons.

## Conclusion

Interneurons are an essential part in brain tissue oxygen regulation. At least two classes of interneurons (VIP and NOS) can directly produce vasodilation in response to stimulation, and a third can elicit vasoconstriction (SST). Interneurons are good candidates for the control of vasomotion during resting state due to their direct projections on arterioles. The development of interneuron functioning and their projections to vessels should reflect the development of neuronal response in order to prevent localized hypoxia. The interference with normal maturation processes early in development may affect NOS interneuron neurovascular interactions to a greater degree, while insults later in development may be more targeted toward VIP- and SST-mediated mechanisms of oxygen regulation. Importantly, these differences in development also point to age-related pathological onset of various psychiatric disorders, such as schizophrenia and anxiety disorders (Kim-Cohen et al., 2003; Dalsgaard et al., 2020; Collins and Ninan, 2021), as well as the development of learning deficits following neonatal anesthesia exposure (Gascoigne et al., 2021, 2022). Research in this field is ongoing and will likely be able to address interneuron subtype specific contributions to a range of pathologies.

## Author contributions

DA and DG: conceptualization and roles/writing—original draft. DA and AD: funding acquisition and supervision. DA: project administration and resources. JD and AD: writing—review and editing. All authors contributed to the article and approved the submitted version.
